# Active Films of Cassava Starch Incorporated with Carvacrol Nanocapsules

**DOI:** 10.3390/foods13081141

**Published:** 2024-04-09

**Authors:** Aline Krümmel, Carlos Henrique Pagno, Patrícia da Silva Malheiros

**Affiliations:** 1 Laboratory of Microbiology and Food Hygiene, Institute of Food Science and Technology, Federal University of Rio Grande do Sul (UFRGS), Porto Alegre 91501-970, Brazil; krummelaline@gmail.com; 2 Laboratory of Phenolic Compounds, Institute of Food Science and Technology, Federal University of Rio Grande do Sul (UFRGS), Porto Alegre 91501-970, Brazil; cpagno@gmail.com

**Keywords:** active packaging, essential oils, food safety, nanotechnology, natural antimicrobials

## Abstract

The synthesis of active films with natural antimicrobials from renewable sources offers an alternative to conventional non-biodegradable packaging and synthetic additives. This study aimed to develop cassava starch films with antimicrobial activity by incorporating either free carvacrol or chia mucilage nanocapsules loaded with carvacrol (CMNC) and assess their impact on the physical, mechanical, and barrier properties of the films, as well as their efficacy against foodborne pathogens. The addition of free carvacrol led to a reduction in mechanical properties due to its hydrophobic nature and limited interaction with the polymeric matrix. Conversely, CMNC enhanced elongation at break and reduced light transmission, with a more uniform distribution in the polymeric matrix. Films containing 8% carvacrol exhibited inhibitory effects against *Salmonella* and *Listeria monocytogenes*, further potentiated when encapsulated in chia mucilage nanocapsules. These findings suggest that such films hold promise as active packaging materials to inhibit bacterial growth, ensuring food safety and extending shelf life.

## 1. Introduction

The susceptibility of food to the presence and multiplication of microorganisms poses a challenging issue for the industry in maintaining food safety and quality until consumption. An important concern is that, in addition to the modification of organoleptic characteristics caused by spoilage microorganisms, food can also be a source of disease when contaminated by pathogenic microorganisms and their toxins [1]. An alternative adopted by the food industry is the use of chemical preservatives, aiming to protect against pathogenic microorganisms and extend the shelf life of products. However, the ingestion of excessive synthetic additives by the population can result in adverse health effects [2]. In this context, natural preservatives have been the target of numerous recent studies as potential substitutes for synthetic preservatives [3,4,5,6,7,8,9]. It is essential to note that, while many natural compounds offer potential benefits, their safety for human consumption can vary depending on factors such as concentration. Therefore, it is crucial to prioritize Generally Recognized as Safe (GRAS) compounds or conduct thorough toxicity assessments to ensure their safety and suitability for use.

Antimicrobial additives from natural sources, such as plant essential oils, are a promising alternative for the food industry, due to their availability, low toxicity, and sustainable nature. Carvacrol, present in oregano and thyme essential oils, stands out as a phenolic compound with significant antioxidant and antimicrobial activity, acting against Gram-positive and Gram-negative pathogenic bacteria, filamentous fungi, and yeasts [8], and is categorized as GRAS by the Food and Drug Administration (FDA). However, it is highly susceptible to processing and storage, due to exposure to heat, light, or oxygen, representing a major challenge for its effective incorporation into food formulations [9].

One strategy to overcome this problem is encapsulation, which aims to establish a physical barrier that protects the encapsulated compound from environmental conditions, in addition to controlling its release at specific rates and locations [10]. The encapsulation technique enables the retention of a sensitive compound, referred to as the core, within a nanoparticle produced from a polymeric matrix, known as the wall material, providing a progressive release of the encapsulated compound [11]. The core material can be protected against adverse food processing conditions, such as a high temperature, humidity, and extreme pH, among others, while also masking undesirable odors and improving the bioactivity, solubility, and stability of the encapsulated compounds [12].

The polymers used as wall material can be synthetic or natural. Polysaccharides such as chitosan, alginate, starch, pectin, gums, and mucilages are natural polymers frequently used in the nanoencapsulation of oils [13]. Mucilages have desirable properties, such as a high hydration capacity, stability, non-toxicity, and low cost, and, therefore, stand out when compared to other natural polymers. Different mucilages extracted from edible vegetables, such as basil, flaxseed, and chia seeds, are being studied for their use as wall materials in nanoparticles [14,15,16,17]. Chia (*Salvia hispanica* L.) is native to Mexico and Guatemala [18], belongs to the *Lamiaceae* family, and is a vegetable that has been recognized by the Food and Agricultural Organization (FAO) since 1996 as a potential source of mucilage, due to its excellent gel-forming properties in aqueous solutions, even at low concentrations [19]. Furthermore, chia mucilage shows antimicrobial activity against Enterobacteriaceae [20], *Salmonella enterica* sv. Typhimurium, and *Campylobacter jejuni* [21].

There is a growing emphasis on sustainability in the packaging production landscape, prompting increased research and advancements in alternative materials aligned with ecologically responsible principles [22]. The goal is to reduce the consumption of non-biodegradable polymers derived from petroleum-based products, which directly contribute to environmental pollution. Biodegradable films obtained from renewable sources are an alternative to mitigate the environmental impact of traditional packaging materials, most of which are derived from non-renewable sources. Biopolymers such as proteins (gelatin, casein, caseinates, ovalbumin, wheat gluten, and zein), polysaccharides (starch, chitosan, pectin, and cellulose), and lipids (acetylated monoglycerides, waxes, and fatty acid esters) stand out as the main sources for the manufacturing of biodegradable packaging [23,24]. The specific choice of cassava starch as a polymeric matrix for the development of films in this study is based on its abundant availability, affordable cost, and inherent biodegradation capacity, in line with the growing demands for sustainable solutions in the packaging industry [8,25]. In addition to the possibility of developing biodegradable formulations, packaging can also interact with food, in which case, it is referred to as active packaging. There are different types on the market depending on the desired application, including antimicrobial packaging, antioxidants, a controlled atmosphere, and moisture barriers, among others [26,27]. Active packaging containing antimicrobial agents and natural antioxidants is a great option for extending the shelf life of food products [28].

To sustainably maintain food safety and quality while reducing or replacing synthetic preservatives that may pose health risks to consumers, the development of active packaging containing natural antimicrobials is a promising solution. However, the addition of carvacrol nanocapsules to biodegradable films remains largely unexplored. This study aimed to contribute to the scientific knowledge on active packaging by developing cassava starch films with antimicrobial properties through the incorporation of free carvacrol or chia mucilage nanocapsules loaded with carvacrol (CMNC), and to evaluate the impacts of these natural antimicrobials on the physical, mechanical, and barrier properties of the films.

## 2. Materials and Methods

### 2.1. Materials

Cassava starch (Yoki Alimentos, São Paulo, Brazil) was used as the basis to produce the films and glycerol (Merk, São Paulo, Brazil) as a plasticizer. Chia seeds (*Salvia hispanica* L., in bulk) were purchased from a local market (Porto Alegre, Brazil). The seeds were stored in vacuum-sealed bags until further use. Ethanol and Tween 80 were acquired from Dinamica (São Paulo, Brazil). Carvacrol (98%) was acquired from Sigma-Aldrich (St. Louis, MO, USA). All other reagents used were of analytical grade.

### 2.2. Bacterial Cultures

To determine antimicrobial activity, bacterial inoculation cocktails were prepared, each comprising three strains, following the methodology described by Cacciatore et al. [29]. The *Listeria monocytogenes* cocktail was formed by *L. monocytogenes* 7459, *L. monocytogenes* J11, and *L. monocytogenes* ATCC 7644, while the *Salmonella enterica* cocktail was formed by *S.* Enteritidis SE86, *S.* Minnesota 7301007, and *S.* Heidelberg 22295. The strains were cultured separately in Brain Heart Infusion (BHI) broth at 37 °C for 18 h. Next, each strain was adjusted to a concentration of 8 log CFU/mL by adding BHI broth until an optical density (OD_630nm_) of approximately 0.5 was reached using an Ultrospec™ 3100 Pro spectrophotometer (GE Healthcare, Amersham, UK). Subsequently, 3 mL of each strain was added to a single tube, resulting in 9 mL of inoculant cocktails, with bacterial suspensions prepared immediately before each test. All strains used in this study were isolated from food or surfaces and belong to the Microbiology and Food Control Laboratory (ICTA/UFRGS, Porto Alegre, Brazil).

### 2.3. Chia Mucilage Extraction

Chia mucilage was obtained using the methodology proposed by Dick et al. [30], with some modifications. Chia seeds were immersed in water at a ratio of 1:30 (*w*/*v*) and subjected to agitation using a mechanical propeller shaker (Edutec, EEQ9008A-2, Curitiba, Brazil) for 2 h at room temperature. Then, the mucilage formed was separated from the chia seeds by centrifugation (HITACHI High-Speed Refrigerated, model CR21GIII, Ibaraki, Japan) at 9000× *g* for 30 min. Soon after, the mucilage firmly adhering to the seeds was separated with the aid of a sieve and a vacuum pump. Following this, the mucilage underwent filtration using a fine mesh to eliminate small particles. Finally, it was dried in an oven with forced air circulation (DeLeo, model B5AFD, Porto Alegre, Brazil) at 60 °C for 18 h and then stored in vacuum-sealed plastic packaging until use.

### 2.4. Production of Chia Mucilage-Based Nanocapsules Loaded with Carvacrol and Carvacrol Emulsion

Nanocapsules containing carvacrol were produced with chia mucilage, according to the methodology described by Campo et al. [16]. The amount of each component to be added was determined in previous studies [31]. Briefly, an organic phase and an aqueous phase were prepared for encapsulation. The organic phase containing Tween 80 (13.5 mg), carvacrol (40 mg), and ethanol (4 mL) was maintained under magnetic stirring for 15 min. The aqueous phase was prepared with chia mucilage hydrated in distilled water (0.1% *w*/*v*), mixed with a magnetic stirrer for 2 h at room temperature (25 °C), and autoclaved (121 °C for 15 min). The organic phase was added dropwise to 20 mL of the aqueous phase during homogenization in Ultra-Turrax (digital model T25; IKA, Staufen, Germany), thus forming the nanoparticles (final carvacrol concentration of 1.67 mg/mL). Previous work conducted by our research group revealed that the diameter size of CMNC was approximately 179 nm, with a zeta potential of −11.4 mV and an encapsulation efficiency of 98.65% [31].

Additionally, a carvacrol emulsion, referred to as free carvacrol in this work, was prepared by vortex homogenizing 10 mL of sterile distilled water, 0.106 g of carvacrol, and 0.1 g of Tween 80 [29].

### 2.5. Film Preparation

The biodegradable films were prepared using the casting technique, following the methodology proposed in previous studies by Assis et al. [32], with some modifications. The film-forming solution was prepared with 4% cassava starch (Yoki Alimentos, São Paulo, Brazil) in distilled water (4 g/100 g of solution). The solution was gelatinized in a water bath at 80 °C for 20 min under constant stirring. After starch gelatinization, glycerol was then added (0.25 g/g starch). Free or nanoencapsulated carvacrol was incorporated at concentrations of 2%, 5%, and 8% (*v*/*v*) into the film-forming solution at 35 °C, as determined in a previous study [32,33]. The film-forming solution was then placed in polystyrene Petri dishes (0.30 g/cm^2^) and dried in an oven with forced air circulation at 35 °C for 16 h. A control film made of cassava starch without the addition of any antimicrobial agent was also prepared.

### 2.6. Thickness and Mechanical Properties of the Films

The thickness of the films was measured using a digital micrometer (Digimess, IP40, São Paulo, Brazil) at random points, with the average of five readings reported. The mechanical properties were assessed using a texturometer (Stable Micro Systems, TA.XT2i, Godalming, UK) equipped with a 5 kg load cell, an initial distance between claws of 50 mm, and a traction speed of 0.8 mm/s. The tensile strength (TS, MPa) and elongation at break (E, %) were determined for ten strips of each film (100 × 25 mm), according to the methodology described in ASTM D882-09 [34].

### 2.7. Water Vapor Permeability (WVP)

The water vapor permeability of the films was evaluated according to the standard method of the American Society for Testing and Materials (ASTM 96-05) described by Pagno et al. [35]. The films were fixed in aluminum permeation cells with internal dimensions of 63 mm in diameter and 25 mm in height, containing granular anhydrous CaCl_2_. These cells were then placed in a glass vat with a relative humidity of 75% and maintained at 25 °C, with a relative humidity gradient of 0/75% achieved through a saturated NaCl solution. After 24 h, the weight gain of each permeation cell set was measured using an analytical balance. Water vapor permeability was calculated using the following equation:

(1) $$WVP=\frac{w.\;L}{A.\;t.\;∆p\;}\;$$

where *w* is the weight of water permeated through the film (g), *L* is the thickness of the film (mm), *A* is the permeation area (m^2^), *t* is the time of permeation (h), and Δ*p* is the water vapor pressure difference between the two sides of the film (KPa).

### 2.8. Moisture and Solubility in Water

The moisture content and water solubility of the biodegradable films were determined according to the method described by Carissimi et al. [36]. To determine humidity, the samples were cut (2 cm diameter discs), weighed, and dried in an oven with air circulation and renewal (105 °C—24 h). After the drying and weighing process, the samples were added with 30 mL of distilled water and shaken for 24 h at 25 °C. After this step, the water in the container was removed and filtered. The non-solubilized samples were dried at 105 °C for 24 h. Solubility was determined according to the equation:

(2) $$S\;\%=\frac{Wi-Wf}{Wi}\times 100$$

where *Wi* is the initial dry weight of the sample (g) and *Wf* is the final dry weight (g).

### 2.9. Optical Properties

The color of the films was evaluated using a Minolta colorimeter (model CR-300; Minolta, Osaka, Japan) according to the CIELab scale, where L* represents luminosity, ranging from 0 (black) to 100 (white); a* varies from green (−) to red (+); and b* varies from blue (−) to yellow (+). A white disc (*L*_0_: 98,05, *a*_0_: 0.14, and *b*_0_: 0.74) was used as a standard. The color difference compared to white (∆E) was calculated by Equation (3) [9]:

(3) $$∆E=\sqrt{{{(∆L}^{*})}^{2}+{{(∆a}^{*})}^{2}+{{(∆b}^{*})}^{2}\;}$$

where: ΔL* = L* − *L*_0_; Δa* = a* − *a*_0_; Δb* = b* − *b*_0_.

Δ*E* is a standard measurement, created by the Commission Internationale de l’Eclairage (International Commission on Illumination), that quantifies the difference between two colors displayed on a screen. Its levels are the difference between the displayed color and the original color standard of the input content. Lower Δ*E* figures indicate a greater accuracy, while high Δ*E* levels indicate a significant mismatch. The measurement scale of this parameter varies from 0 to 100, where 0 is a smaller color difference and 100 indicates complete distortion, with the following perception ranges [37]:

≤1.0: Not perceptible by the human eye

1–2: Perceptible through close observation

2–10: Perceptible at a glance

11–49: Colors are more similar than opposite

100: Colors are exactly opposite

The opacity of the films was determined based on the methodology proposed by Tunç e Duman [38]. Film rectangles (2 × 1 cm) were cut and inserted directly into a quartz cuvette, and then the absorbance was measured using a spectrophotometer (Shimadzu, UV-1800, Kyoto, Japan) in the range from 210 nm (UV light region) to 600 nm (visible light region). An empty cuvette was used as a reference. The experiment was carried out in duplicate, with readings taken at a wavelength of 600 nm. The opacity (Op) of the films was calculated according to the equation [35]:

(4) $$Op=\frac{{Abs}_{600}}{x}$$

where *Abs*_600_ is the absorbance value at 600 nm and *x* is the film thickness (mm).

### 2.10. Morphological Properties

The morphology of the previously selected films was evaluated using a scanning electron microscope (JEOL, JSM 6060, Tokyo, Japan). The samples were cut into small pieces and attached with double-sided tape to aluminum stubs. All samples were metallized with a thin layer of gold, in the range of 20 nm for 100 s at a current of 40 mA. The surface and cross-section were observed with an acceleration voltage of 5 kV and a magnification of 500× [32].

### 2.11. Thermogravimetric Analysis

The thermal stability of the selected films was analyzed using a Shimadzu Instrument (model TGA-50). Samples of approximately 5 mg were heated from 25 °C to 650 °C at a heating rate of 10 °C/min under a nitrogen flow. Stability was analyzed by weight loss versus temperature.

### 2.12. Antimicrobial Properties of the Films

The in vitro antimicrobial activity of the films was assessed using the viable cell counting method, as proposed by Liao et al. [39]. The day before the experiment, the bacteria inoculum was prepared in BHI broth and incubated in an oven at 37 °C overnight. The bacterial concentration was then adjusted to 10^6^ CFU/mL using BHI broth. For the test, rectangles of film (2 × 1 cm), previously sterilized in UV light for 15 min on both sides, were placed in an Eppendorf tube containing 800 µL of the bacterial suspension (10^6^ CFU/mL). The tubes were incubated in a B.O.D incubator at 25 °C for 24 h under constant shaking. After incubation, successive dilutions were made in 0.1% peptone water. Each dilution was then plated using the microdrop technique (20 µL). Xylose Lysine Deoxycholate Agar (XLD) (Kasvi, Paraná, Brazil) was used for *Salmonella*, while BHI Agar (Sigma-Aldrich, St. Louis, MO, USA) was used for *Listeria monocytogenes*. The plates were incubated at 37 °C for 18 h. The number of viable cells was then manually counted and multiplied by the dilution factor. The microbial counts are reported as log_10_ CFU/g.

### 2.13. Statistical Analyses

Data analysis was performed using the Statistica 12.0 software (StatSoft, Inc, Tulsa, OK, USA) through analysis of variance (ANOVA) followed by Tukey’s test, with a confidence level of 95%. All experiments were conducted in triplicate, and the results are presented as mean ± standard deviation.

## 3. Results and Discussion

### 3.1. Thickness and Mechanical Properties

**Figure 1:**
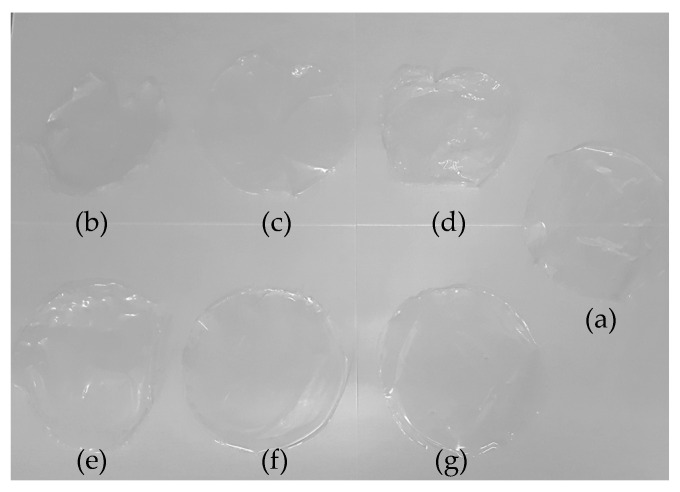
Visual appearance of cassava starch films with carvacrol free or chia mucilage nanocapsules loaded with carvacrol: (**a**) CSF—cassava starch films with glycerol; (**b**) C2%; (**c**) C5%; and (**d**) C8%; and (**e**) CMNC2%; (**f**) CMNC5%; and (**g**) CMNC8%.

Active cassava starch films added with 2%, 5%, and 8% of free carvacrol (C2%, C5%, and C8%) or chia mucilage nanocapsules loaded with carvacrol (CMNC2%, CMNC5%, and CMNC8%) are shown in Figure 1.

The thickness of the films varied from 0.12 mm to 0.18 mm, with significant differences (*p* < 0.05) observed between the formulations (Table 1). Increasing the carvacrol concentration did not result in significant changes in the film thickness. Nevertheless, lower uniformity was observed in the films with the addition of free carvacrol compared to those containing CMNC and the control group. The thickness of the films produced in relation to the control film was not significantly changed by the addition of free or nanoencapsulated carvacrol. However, an increase in film thickness was observed with the incorporation of carvacrol nanocapsules, which can be attributed to the increase in the solids content added to the polymer matrix. A similar effect was observed in a previous study [8], where the addition of lycopene nanocapsules increased the thickness of cassava starch films, varying between 0.123 mm and 0.152 mm for the control film and the film with a higher concentration of nanocapsules, respectively.

The incorporation of free or nanoencapsulated carvacrol resulted in a significant (*p* < 0.05) increase in the tensile strength (TS) of the films compared to the standard CSF film. This increase in tension was directly correlated with the increase in force required to break the films. This behavior was due to changes in the interactions that occur, mainly due to the increase in hydrogen bonds [40].

The elongation at break (E%) decreased significantly when free carvacrol was incorporated into the film formulations, regardless of the concentration tested. Previous studies support this finding and infer that the incorporation of a hydrophobic compound, such as an essential oil, into a hydrophilic matrix can promote the formation of discontinuities in the structure, thereby reducing the elasticity of the films [8,9,41,42].

A significant increase in E% was observed, compared to the control films and films with the addition of free carvacrol, when CMNC was incorporated into the film (*p* < 0.05). This enhancement may have been related to the encapsulation of the antimicrobial compound, along with the presence of a surfactant and plasticizer in its composition. These components facilitate the solubility of hydrophobic compounds in water and reduce incompatibility with the polymeric matrix. Similar behavior was observed in a previous study [32], where the addition of β-carotene nanocapsules to biodegradable films on cassava starch resulted in a greater elongation at break compared to the control film and a film with the addition of free β-carotene, at all concentrations studied.

### 3.2. Water Vapor Permeability (WVP), Moisture Content (MC), and Water Solubility (WS)

The results of the water vapor permeability, moisture content, and water solubility tests of the films containing 2%, 5%, and 8% of free or nanoencapsulated carvacrol, as well as the control film, are presented in Table 2.

The permeability of the biodegradable films ranged from 0.34 ± 0.03 g mm m^−2^ h^−1^ kPa^−1^ to 0.53 ± 0.05 g mm m^−2^ h^−1^ kPa^−1^. The addition of carvacrol nanocapsules did not affect the water vapor permeability in comparison to the control film (CSF). However, formulations containing free carvacrol showed significant differences (*p* < 0.05), resulting in an increase in this property. However, increasing the concentration did not affect this parameter in any of the formulations tested. It is inferred that this result may have been associated with the microcracks formed in the films obtained with the addition of free carvacrol. Additionally, the presence of micropores on the film surface further suggested an increased permeability, a phenomenon observed in similar previous studies [8,9].

The moisture content of the films ranged from 10.16 ± 0.14% to 14.67 ± 0.86%, with the films supplemented with CMNC showing a higher moisture content when compared to the films with free carvacrol and the control film. In general, nanocapsules have a greater affinity for water and result in films with a higher moisture content, which can result in increased permeability to water vapor [32,43]. The moisture content of the films decreased significantly (*p* < 0.05) with the presence of free carvacrol and reduced with an increasing concentration.

Regarding the solubility of the films in water, the results obtained in this study (Table 2) are in a range similar to the value reported by a previous study [32], which observed a solubility of 17% for cassava starch films (control). A significant difference (*p* < 0.05) was observed between the treatments studied, indicating that the addition of free or nanoencapsulated carcavrol in the proportions studied influenced this parameter. The incorporation of free or nanoencapsulated carvacrol in the film formulations resulted in a decrease in solubility, representing a significant advantage for the development of packaging aimed at preserving foods with a high water content and requiring the controlled release of antimicrobials or antioxidants.

### 3.3. Optical Properties of the Films

Table 3 shows the results of the color and opacity parameters of the films. The L* and ΔE* values show that there was no significant difference between the formulations for these parameters, while the addition of the free or nanoencapsulated compound led to a change in color (*p* < 0.05), with a decrease in a* and an increase in b* compared to the CSF control film. Regarding the ΔE parameter, the results varied from 2.29 to 3.36, which is in the range between 2 and 10, thus characterizing a color difference that is perceptible at first glance, in accordance with the perception ranges described in the literature [37].

In the presence of carvacrol, the films remained transparent (Figure 1, Table 2). The opacity of the films increased from 0.42 A.mm^−1^ without carvacrol to 0.90 and 0.58 A.mm^−1^ with the incorporation of free and nanoencapsulated carvacrol, respectively, at the highest concentration tested in this study (8%). This change, however, was not visually apparent. The yellowish color, characteristic of carvacrol, may have been responsible for this increase in opacity. The low opacity values may be an indication of the good dispersion of carvacrol in the polymer matrix, as the presence of a non-miscible dispersed phase and the type of intermolecular interaction can promote opacity, changing the refractive index and the amount of light passing through the film [44,45].

### 3.4. Morphological Properties

Based on the results obtained in the previous tests presented, films from the 8% formulations were selected to proceed with the analyses.

**Figure 2:**
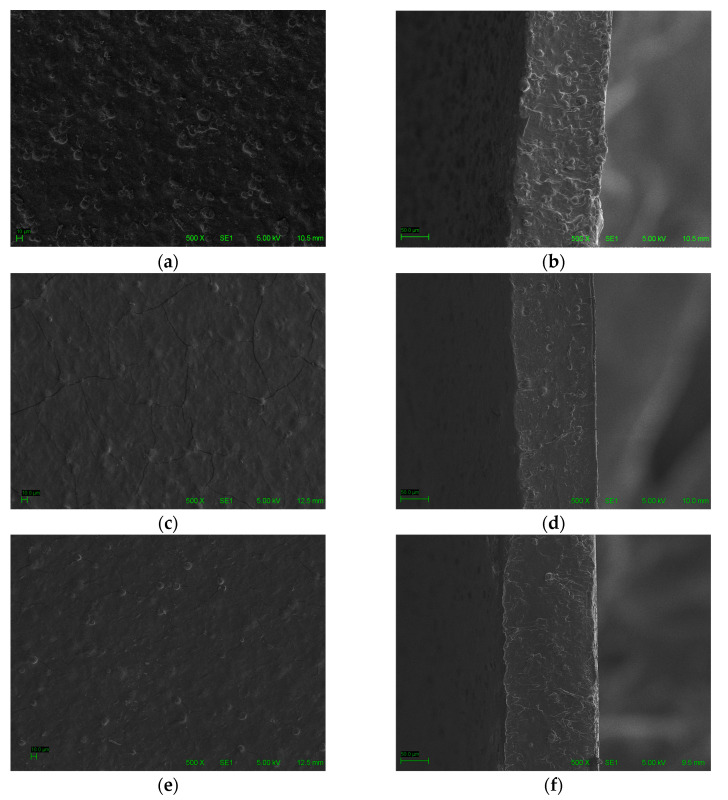
Scanning electron microscopy of the films: (**a**) CSF surface; (**b**) cross-section of the CSF; (**c**) film surface of C8%; (**d**) film cross-section of C8%; (**e**) film surface of CMNC8%; and (**f**) film cross-section of CMNC8%.

The scanning electron microscopy of the surfaces and cross-sections of the CSF, C8%. and CMCN8% films is shown in Figure 2.

The control film (CSF) presented a cohesive structure without the presence of cracks along the matrix. However, the addition of carvacrol of a hydrophobic nature resulted in the formation of heterogeneous films with the presence of microcracks throughout their structure (Figure 2c). This outcome can be attributed to the random distribution of the hydrophobic antimicrobial, which reduced its affinity with the hydrophilic matrix. Consequently, this justifies the observed decrease in film elasticity upon the addition of free carvacrol compared to other films.

Similar findings were previously reported [46] in cassava starch films added with rosemary extract, in which an increase in the concentration of the extract led to films with cracks and less homogeneity, and it was also found that the reduced interaction between the antioxidant additive and the matrix resulted in a decrease in the mechanical properties of the films. The appearance of cracks in the film supplemented with free carvacrol corroborates the results obtained in this study for water vapor permeability (WVP), explaining the increase in the value related to this formulation.

The presence of carvacrol nanocapsules in the formulation resulted in more homogeneous films without large agglomerates and no cracks in their structure, demonstrating a greater affinity of the nanoencapsulated antimicrobial with the polymeric matrix. The presence of pores in the film structure justifies the increase in the water vapor permeability (WVP) of the films of this formulation, when compared to the control film, but it was still lower (*p* < 0.05) than that of the film supplemented with free carvacrol.

### 3.5. Thermogravimetric Analysis

**Figure 3:**
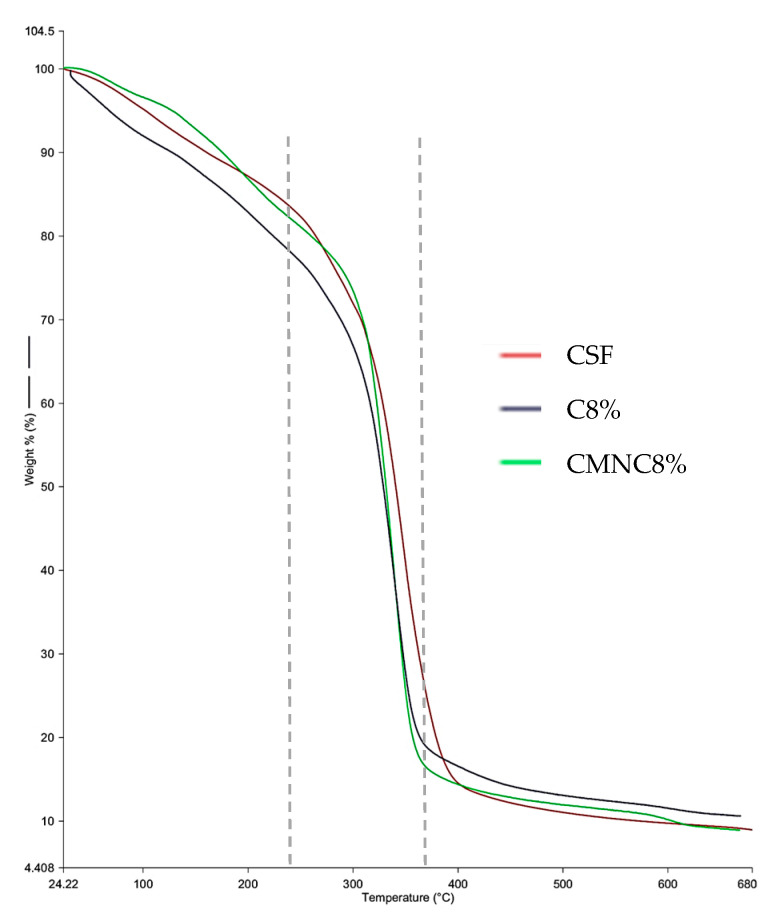
Thermogravimetric analysis (TGA) of the control film, 8% carvacrol free film, and film with 8% chia mucilage nanocapsules loaded with carvacrol.

A thermogravimetric analysis (TGA) was performed on the control films, the film with 8% free carvacrol, and the film with 8% CMNC, and their thermal degradation curves are shown in Figure 3.

The TGA showed the mass loss of biodegradable films upon heating. Regardless of whether the antimicrobial was added in free form or nanoencapsulated, the films exhibited similar behavior to the control film, with three stages of mass loss, as described in the literature for the thermal stability of starch films with the addition of glycerol as a plasticizer. The dashed lines in the figure delineate the stages of film degradation. The first stage, in the range of 100 °C, corresponded to the loss of residual moisture present in the film following the production process. The second stage occurred in the temperature range of 200 °C and was characterized by the decomposition of glycerol, the beginning of starch decomposition, the volatilization of carvacrol, whose boiling temperature is 238 °C [47,48], and the loss of the low-molecular-weight fraction or water associated with the polymeric matrix [32,42,49]. The third stage was from 350 °C, resulting in a residual mass of approximately 9% for the starch (CSF) and CL8% films and approximately 11% for the NC8% film.

Similar results were obtained for biodegradable films based on cassava starch containing free and nanoencapsulated β-carotene [32], as well as for active quinoa films incorporated with oregano and thyme essential oils [35], and also for bioactive gelatin films containing silver nanoparticles [50].

In general, the casting methodology used in this study allowed for the production of cassava starch films with good characteristics. All the films produced, both with free and nanoencapsulated carvacrol, showed adequate properties. Cassava starch has proven to be a good source for obtaining uniform and biodegradable films [8,9,26,32,46,49]. The presence of carvacrol did not affect the thickness of the films, which remained transparent and had physical and thermal characteristics suitable for use as food packaging. These results are in agreement with previous studies [51], where cellulose acetate films with the addition of carvacrol were investigated and it was found that the incorporation of up to 10% of the compound did not cause significant changes in their properties.

### 3.6. Antimicrobial Activity

The antimicrobial activity of the films was evaluated in vitro against the Gram-positive bacteria *Listeria monocytogenes* and Gram-negative *Salmonella*, using the viable cell counting method [39]. *Salmonella* and *L. monocytogenes* are pathogens of significant concern for food safety. *Salmonella* spp. remains one of the primary causes of foodborne outbreaks in Brazil and worldwide [52,53,54]. *Salmonella* infection, known as salmonellosis, manifests with symptoms such as fever, abdominal pain, diarrhea, nausea, and vomiting. Severe dehydration resulting from these symptoms may require hospitalization, especially among older individuals and those with compromised immune systems, where the disease can be fatal [54]. *Listeria monocytogenes*, an emerging pathogen, has been implicated in numerous foodborne outbreaks worldwide in recent years. Listeriosis, the disease caused by this bacterium, is associated with a high rate of hospitalization and mortality (20 to 40% of cases) and poses a significant risk to vulnerable populations such as older people, immunocompromised individuals, and pregnant women and their infants [53,54]. Initial symptoms typically include muscle pain, vomiting, and diarrhea, which may progress to more severe conditions such as meningitis, septicemia, and other neurological disorders. In pregnant women, listeriosis can result in miscarriage [54].

**Figure 4:**
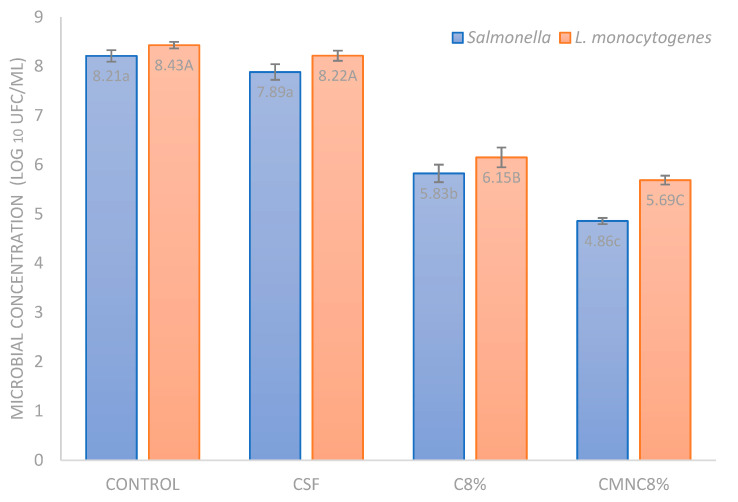
Microbial counts of *Salmonella* and *L. monocytogenes* after 24 h of incubation in contact with cassava starch films with and without carvacrol compared to a control without film. Top bars represent standard deviation of means. Different letters in the same color bar indicate a significant difference (*p* < 0.05). CONTROL: tube with bacteria only (without film); CSF: bacteria + cassava starch film; C8%: bacteria + film with 8% free carvacrol; and CMNC8%: bacteria + film with chia mucilage nanocapsules loaded with carvacrol.

Figure 4 shows the antimicrobial effect of the cassava starch films produced with 8% free carvacrol, 8% CMNC, and carvacrol-free, compared to a control without film. Except for the starch film (CSF), which demonstrated no antimicrobial activity, all other films investigated in this study showed inhibitory effects against the tested bacteria, with a statistically significant difference (*p* < 0.05). Films containing chia mucilage nanocapsules loaded with carvacrol (CMNC8%) showed higher inhibition rates. These films exhibited reductions of 3.35 log CFU/mL for *Salmonella* and 2.74 log CFU/mL for *L. monocytogenes*, surpassing the reduction observed in the films with free carvacrol (C8%), which showed reductions of 2.39 and 2.28 log CFU/mL for *Salmonella* and *L. monocytogenes*, respectively. These results suggest that carvacrol nanoencapsulation provides protection and controlled release of the compound, increasing the antimicrobial efficacy of the films. These findings are promising for the development of active packaging with antimicrobial properties.

Other researchers have also obtained promising results, demonstrating that both free carvacrol and carvacrol incorporated into cellulose acetate films effectively inhibited the tested bacteria, *Weissella viridescens* and *Pseudomonas fluorescens* [51]. In a related study [55], initial populations of *Salmonella* Enteritidis and *Listeria monocytogenes* equal to 6.24 and 6.84 log CFU/mL, respectively, decreased to 4.72, 5.14, and 4.50 log CFU/mL for *S.* Enteritidis after 12 h in protein films (CP) containing marjoram (MA), coriander (CO), and clove (CL) essential oil, respectively. The reduction in the population of *L. monocytogenes* also ranged from 1 to 2 log CFU/mL within the same time frame, with protein films containing CO or CL demonstrating a greater effectiveness against *L. monocytogenes* compared to CP-MA films.

It was possible to verify that the cassava starch films produced in the present study, with glycerol as a plasticizer and incorporated with free or nanoencapsulated carvacrol, exhibited antimicrobial activity against Gram-positive and Gram-negative bacteria, a fact that was also noticed in other research [55,56] when studying the antibacterial action of active films. Studies carried out with carvacrol have shown a broad spectrum of antimicrobial activity against Gram-positive and Gram-negative bacteria [47,57,58].

The control film did not exhibit antimicrobial activity against any of the pathogens tested, which has also been widely observed by the scientific community when evaluating the antimicrobial activity of starch films [35,59,60,61,62,63].

## 4. Conclusions

Carvacrol emerges as a promising natural antimicrobial agent for active packaging formulations, with encapsulation in chia mucilage nanocapsules enhancing its antimicrobial efficacy. Chia mucilage nanocapsules loaded with carvacrol exhibited a greater compatibility with the polymeric matrix, resulting in improved physical–chemical and mechanical properties of the films compared to free antimicrobials, providing the films with a higher elongation at break and homogeneous distribution in the polymeric matrix without altering the stability thermal properties of the films. These findings are important for the development of active packaging with enhanced flexibility and efficacy. Moreover, the results showed that films incorporating 8% carvacrol exerted significant inhibitory effects against *Salmonella* and *Listeria monocytogenes*, suggesting their potential as active packaging materials for inhibiting bacterial growth. Considering the promising results obtained in the present study and the fact that encapsulation can increase the stability of carvacrol while masking its pronounced odor, future perspectives include applying the films in a food matrix and conducting stability studies on the films. Additionally, investigating possible organoleptic interferences with different film formulations in food will be essential to evaluate their viability for future market applications.

## Figures and Tables

**Table 1:** Thickness, tensile strength (TS), and elongation at break (E) of films with free carvacrol (C) and chia mucilage nanocapsules loaded with carvacrol (CMNCs).

Sample	Thickness (mm)	TS (MPa)	E (%)
CSF	0.15 ± 0.02 ^ab^	0.73 ± 0.06 ^b^	191.14 ± 10.89 ^b^
C2%	0.12 ± 0.03 ^b^	1.32 ± 0.34 ^a^	157.82 ± 11.92 ^c^
C5%	0.14 ± 0.02 ^b^	1.33 ± 0.46 ^a^	156.51 ± 12.37 ^c^
C8%	0.13 ± 0.03 ^b^	1.34 ± 0.51 ^a^	157.98 ± 11.58 ^c^
CMNC2%	0.17 ± 0.01 ^a^	1.31 ± 0.11 ^a^	264.89 ± 13.01 ^a^
CMNC5%	0.17 ± 0.01 ^a^	1.30 ± 0.23 ^a^	265.54 ± 12.93 ^a^
CMNC8%	0.18 ± 0.02 ^a^	1.31 ± 0.17 ^a^	265.10 ± 13.56 ^a^

Values are expressed as mean ± standard deviation. Different letters in the same column indicate significant differences (*p* < 0.05). CSF: cassava starch films with glycerol; C2%: films with 2% free carvacrol; C5%: films with 5% free carvacrol; C8%: films with 8% free carvacrol; CMNC2%: films with 2% chia mucilage nanocapsules loaded with carvacrol; CMNC5%: films with 5% chia mucilage nanocapsules loaded with carvacrol; and CMNC8%: films with 8% chia mucilage nanocapsules loaded with carvacrol.

**Table 2:** Water vapor permeability (WVP), moisture content (MC), and water solubility (WS) of films with free carvacrol and carvacrol nanocapsules.

Sample	WVP (g mm m^−2^ h^−1^ kPa^−1^)	MC (%)	WS (%)
CSF	0.34 ± 0.03 ^b^	13.48 ± 0.22 ^b^	17.89 ± 0.42 ^a^
C2%	0.52 ± 0.05 ^a^	11.16 ± 0.34 ^c^	16.12 ± 0.29 ^b^
C5%	0.53 ± 0.05 ^a^	11.37 ± 0.25 ^c^	15.98 ± 0.33 ^b^
C8%	0.52 ± 0.06 ^a^	10.16 ± 0.14 ^d^	14.47 ± 0.76 ^c^
CMCN2%	0.43 ± 0.01 ^ab^	14.67 ± 0.86 ^a^	16.76 ± 0.55 ^b^
CMCN5%	0.43 ± 0.01 ^ab^	14.21 ± 0.33 ^a^	16.38 ± 0.81 ^b^
CMCN8%	0.44 ± 0.02 ^ab^	12.88 ± 0.81 ^b^	15.13 ± 0.64 ^c^

Values are expressed as mean ± standard deviation. Different letters in the same column indicate significant differences (*p* < 0.05). CSF: cassava starch films with glycerol; C2%: films with 2% free carvacrol; C5%: films with 5% free carvacrol; C8%: films with 8% free carvacrol; CMNC2%: films with 2% chia mucilage nanocapsules loaded with carvacrol; CMNC5%: films with 5% chia mucilage nanocapsules loaded with carvacrol; and CMNC8%: films with 8% chia mucilage nanocapsules loaded with carvacrol.

**Table 3:** Color and opacity of biodegradable cassava starch films containing different concentrations of free or nanoencapsulated carvacrol.

Sample	Color Parameters				Opacity (A.mm^−1^)
	L*	a*	b*	∆E*	600 nm
CSF	95.69 ± 0.79 ^a^	0.04 ± 0.01 ^b^	2.53 ± 0.17 ^c^	2.97 ± 0.67 ^a^	0.42 ± 0.07 ^c^
C2%	95.70 ± 0.39 ^a^	−0.07 ± 0.01 ^d^	2.96 ± 0.14 ^a^	3.34 ± 0.36 ^a^	0.79 ± 0.24 ^a^
C5%	96.05 ± 0.12 ^a^	−0.03 ± 0.02 ^c^	2.86 ± 0.04 ^ab^	2.92 ± 0.11 ^a^	0.86 ± 0.10 ^a^
C8%	95.28 ± 1.26 ^a^	0.14 ± 0.03 ^a^	2.65 ± 0.09 ^bc^	3.36 ± 1.06 ^a^	0.90± 0.13 ^a^
CMNC2%	96.79 ± 0.25 ^a^	−0.03 ± 0.01 ^c^	2.65 ± 0.13 ^bc^	2.29 ± 0.24 ^a^	0.51± 0.18 ^b^
CMNC5%	96.21 ± 0.63 ^a^	−0.04 ± 0.02 ^c^	2.63 ± 0.02 ^bc^	2.64 ± 0.58 ^a^	0.57± 0.02 ^b^
CMNC8%	95.86 ± 0.56 ^a^	0.02 ± 0.01 ^b^	2.77 ± 0.16 ^ab^	2.99 ± 0.50 ^a^	0.58± 0.03 ^b^

Values are expressed as mean ± standard deviation. Different letters in the same column indicate significant differences (*p* < 0.05). CSF: cassava starch films with glycerol; C2%: films with 2% free carvacrol; C5%: films with 5% free carvacrol; C8%: films with 8% free carvacrol; CMNC2%: films with 2% chia mucilage nanocapsules loaded with carvacrol; CMNC5%: films with 5% chia mucilage nanocapsules loaded with carvacrol; and CMNC8%: films with 8% chia mucilage nanocapsules loaded with carvacrol.

## Data Availability

The original contributions presented in the study are included in the article, further inquiries can be directed to the corresponding author.
